# [Expression of Concern] Apelin-13 induces autophagy in hepatoma HepG2 cells through ERK1/2 signaling pathway-dependent upregulation of Beclin1

**DOI:** 10.3892/ol.2025.15236

**Published:** 2025-08-20

**Authors:** Qiulin Huang, Xuan Liu, Chao Cao, Junyue Lei, Dong Han, Guodong Chen, Jia Yu, Linxi Chen, Deguan Lv, Zhongyu Li

Oncol Lett 11: 1051–1056, 2016; DOI: 10.3892/ol.2015.3991

Following the publication of this paper, it was drawn to the Editor's attention by a concerned reader that the GAPDH control blots looked unexpectedly similar in Figs. 2C and 3E, in which different experimental conditions were reported, suggesting that an error may have been made in the compilation of the data in one of these figures.

The authors were contacted by the Editorial Office to offer an explanation for this apparent anomaly in the presentation of the data in this paper; however, up to this time, no response from them has been forthcoming. Owing to the fact that the Editorial Office has been made aware of potential issues surrounding the scientific integrity of this paper, we are issuing an Expression of Concern to notify readers of this potential problem while the Editorial Office continues to investigate this matter further.

